# Theoretical and Experimental Substantiation of the Efficiency of Combined-Reinforced Glass Fiber Polymer Composite Concrete Elements in Bending

**DOI:** 10.3390/polym14122324

**Published:** 2022-06-08

**Authors:** Besarion Meskhi, Alexey N. Beskopylny, Sergey A. Stel’makh, Evgenii M. Shcherban’, Levon R. Mailyan, Nikita Beskopylny, Natal’ya Dotsenko

**Affiliations:** 1 Department of Life Safety and Environmental Protection, Faculty of Life Safety and Environmental Engineering, Don State Technical University, Gagarin, 1, 344003 Rostov-on-Don, Russia; spu-02@donstu.ru; 2 Department of Transport Systems, Faculty of Roads and Transport Systems, Don State Technical University, Gagarin, 1, 344003 Rostov-on-Don, Russia; 3 Department of Engineering Geology, Bases, and Foundations, Don State Technical University, 344003 Rostov-on-Don, Russia; sergej.stelmax@mail.ru (S.A.S.); au-geen@mail.ru (E.M.S.); 4 Department of Roads, Don State Technical University, 344003 Rostov-on-Don, Russia; lrm@aaanet.ru; 5 Department Hardware and Software Engineering, Don State Technical University, 344003 Rostov-on-Don, Russia; beskna@yandex.ru; 6 Department of Technological Engineering and Expertise in the Construction Industry, Don State Technical University, 344003 Rostov-on-Don, Russia; natalya_1998_dotsenko@mail.ru

**Keywords:** combined reinforcement, glass fiber polymer composite concrete bending elements, polymer composite reinforcement, ultimate bending moment, compressed zone of concrete

## Abstract

An essential problem of current construction engineering is the search for ways to obtain lightweight building structures with improved characteristics. The relevant way is the use of polymer composite reinforcement and concrete with high classes and prime characteristics. The purpose of this work is the theoretical and experimental substantiation of the effectiveness of combined-reinforced glass fiber polymer composite concrete (GFPCC) bending elements, and new recipe, technological and design solutions. We theoretically and experimentally substantiated the effectiveness of GFPCC bending elements from the point of view of three aspects: prescription, technological and constructive. An improvement in the structure and characteristics of glass fiber-reinforced concrete and GFPCC bending elements of a new type has been proven: the compressive strength of glass fiber-reinforced concrete has been increased up to 20%, and the efficiency of GFPCC bending elements is comparable to the concrete bending elements with steel reinforcement of class A1000 and higher. An improvement in the performance of the design due to the synergistic effect of fiber reinforcement of bending elements in combination with polymer composite reinforcement with rods was revealed. The synergistic effect with optimal recipe and technological parameters is due to the combined effect of dispersed fiber, which strengthens concrete at the micro level, and polymer composite reinforcement, which significantly increases the bearing capacity of the element at the macro level. Analytical dependences of the type of functions of the characteristics of bent concrete structures on the arguments—the parameters of the combined reinforcement with fiber and polymer composite reinforcement—are proposed. The synergistic effect of such a development is described, a new controlled significant coefficient of synergistic efficiency of combined reinforcement is proposed. From an economic point of view, the cost of the developed elements has been reduced and is economically more profitable (up to 300%).

## 1. Introduction

Currently, one of the most relevant areas in construction science and technologies is the search for rational combinations of building materials used, new technologies, and design solutions. The industry of polymer building materials and products is actively developing. An important issue is the search for ways to obtain lightweight building structures as thin as possible and have the smallest sections, while having improved characteristics and structure of the materials used. Considering all the above, the use of polymer composite reinforcement concrete of high class and prime characteristics is relevant and in demand from the point of view of science and practice.

The improvement of reinforced concrete bending elements is the most relevant at the present time and has already been considered in many studies, for example, in [1,2,3,4,5,6,7,8,9,10,11,12,13,14,15]. In this case, various improvement methods are used: reinforcement with polymer composite materials [2,5,8,10,11,12,13,15,16], various fibrous materials [6,9,14], Fe-SMA activation [3], and cathode-ray tubes made of glass waste [7]. The economic aspect was also considered—the cost of reinforced concrete beams largely depends on the brand of reinforcing steel [1].

Using various types of fibers in concrete, the parameters of the distribution of fibers in the body of concrete and the recipe factors for obtaining fiber-reinforced concretes with improved characteristics have been studied in many works [9,17,18,19,20,21,22,23,24,25,26,27]. The effect of fiber on the structure and characteristics of concrete products and structures was studied under the influence of high temperatures [17,21]. In addition, the influence on performing fiber-reinforced concrete of various dosages of fiber [20,25], types of fiber (glass, basalt, polypropylene, steel) [21,22,26], and fiber diameter [24] was also studied in detail. An important technological parameter is the orientation of the fibers and its control in the composite matrix [18].

Research on improving the characteristics and operation parameters of compressed [18,19,28,29,30,31,32,33] and bending elements [6,9,14,17,18,27,33,34,35,36,37,38] because of fiber reinforcement continues to expand and open new possibilities for their application. At the same time, researchers have studied the behavior of columns and beams made of steel fiber concrete [6,30], ultrahigh strength fiber concrete [36], glass geopolymer concrete with hybrid fibers [29], steel fibers [34,35], basalt fibers [34], hybrid epoxy resin, reinforced with flax or fiberglass [28], and steel fibers from scrap tires [31].

Finally, the works of the authors involved in the study of polymer composite concrete bending elements (beams) [2,5,8,11,12,13,15,16,35,37,38,39,40,41,42,43,44,45,46,47,48,49,50] were of particular interest for the development of this study. To improve the performance of bending elements, researchers studied the following for their reinforcement: fiber-reinforced polymers (FRP), glass-reinforced polymers (GFRP), composite polymers, reinforcement with jute–polyester fiber (JPFRP), polymers, reinforcement with basalt fiber (BFRP), carbon (CFRP) and aramid (AFRP) fibers. In this case, polymers were used in the form of rods, sheets, and fibers. The most effective bending elements, both in terms of various properties and in terms of material consumption and economic efficiency, contained complex reinforcement in the form of polymer composite rods and fiber [37,38] as well as steel rods [37]. Such complex reinforcement allows obtaining a greater effect than reinforcement with one of the types of reinforcement.

We previously studied “the quantitative and qualitative aspects of the joint work of fiber in concrete in combination with polymer composite reinforcement, which gave good results in terms of physical and mechanical characteristics”, the most rational design solutions and showed good compatibility between fiber reinforcement and reinforcement with polymer composite rods [51,52]. Detailed information about the polymer composites considered in the study is presented in [53,54,55].

In the study, a numerical calculation and an experimental test were carried out, including an analysis of scanning electron microscopy as well as an economic analysis of the effectiveness of the proposed solutions.

The scientific novelty of the research is the recipe: technological, microstructural and constructive substantiation of the existence of a synergistic effect from the combined reinforcement with dispersed fiber and polymer composite reinforcement of improved bending elements made of heavy concrete. Qualitative and quantitative compositions of concrete, constructive solutions are given, and a mathematical apparatus has been developed to control the effectiveness of the proposed method. Theoretical prerequisites are substantiated from the point of view of the economic efficiency of the developed proposals.

Thus, the purpose of this work is the theoretical and experimental substantiation of the effectiveness of combined-reinforced GFPCC bending elements as well as prescription, technological and design solutions related to the identification of the most rational parameters.

The objectives of the study are:

(1) Determination of rational parameters in terms of formulation and technology;
(2) Obtaining first, glass fiber-reinforced concrete and second, glass fiber polymer composite concrete bending elements of a new type with improved structure and characteristics;
(3) Study from the point of view of theoretical concepts, as well as practical aspects of the structure of such materials and structures, as well as the study of their work from the point of view of experimental test and numerical calculation;
(4) Development of theoretical provisions and substantiation of experimental proposals for the practical industry;
(5) Identifying the most problematic bottlenecks to fill the scientific gap toward joint research between building materials science and building structures, tied to the theory of fiber fibers and polymer composite reinforcing elements.

The results of the study are proposed to be used in the construction of buildings and structures of increased responsibility in relation to extended bending reinforced concrete elements. Thus, the study is relevant in the field of design, construction, and production of building structures, solving the complex problem of the lack of scientific and engineering solutions.

## 2. Materials and Methods

### 2.1. Materials

The study used non-additive Portland cement brand CEM I 52.5N of Novoroscement (Novorossiysk, Russia) GOST 31108-2020 “Common cements. Specifications”. The chemical and mineralogical composition, as well as the physic mechanical properties, are given in [51].

In addition, in the research, granite crushed stone of Pavlovsknerud JSC (Pavlovsk, Russia) and fine aggregate–quartz sand of Quartz Sands LLC (Semenov, Russia) was used. The prime properties of aggregates are presented in [51].

Glass fiber Armplast (Nizhny Novgorod, Russia) was used as dispersed reinforcing fibers, the physical and mechanical characteristics of which are presented in Table 1.

Steel reinforcement was from Tyazhpromarmatura (Aleksin, Russia) according to GOST 34028–2016 “Reinforcing rolled products for reinforced concrete constructions. Specifications” and polymer–composite reinforcement was produced by the Yaroslavl Plant of Composites (Yaroslavl, Russia) according to GOST 31938 “Fiber-reinforced polymer bar for concrete reinforcement. General specifications” [51]. Reinforcement of three diameters was used: 6, 8 and 10 mm. The properties and classes of reinforcement are presented in Table 2.

Detailed information about the polymer composites considered in the study is presented in [53,54,55].

### 2.2. Methods

#### 2.2.1. Numerical Calculation Method

“The calculation was carried out based on SP 63.13330.2018 [58], the manual for the design of concrete and reinforced concrete structures made of heavy concrete without prestressing reinforcement (to SP 52-101-2003)” [59] and the LIRA-SAPR 2016 R5 software (Lira service, Moscow, Russia) [51].

The beam was designed as hinged for concrete classes B30 and B40, steel and composite reinforcement. The length of the beam remained constant, 3000 mm, and was not considered in the calculation of the maximum perceived moment. The calculation of the limiting value of the bending moment was calculated relative to the compressed zone of concrete according to SP 63.13330.2018 p.8.1.8 [58] and the Manual to SP 52.101.2003 p. 3.18 [59]. Four rods were taken for the tensioned bottom reinforcement: the reinforcement of the same material as the bottom one, only with a diameter of 6 mm (smallest diameter) was taken for the compressed reinforcement in the upper zone of the beam.

**Figure 1:**
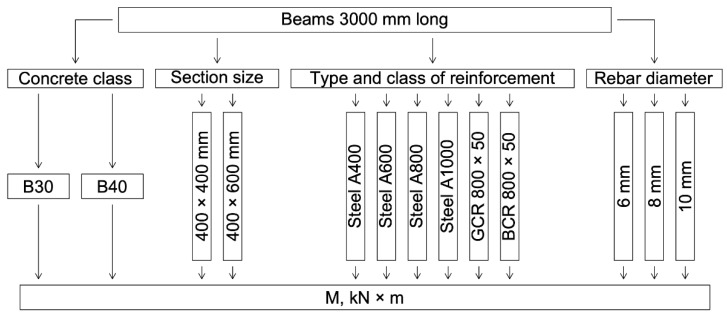
Numerical calculation program.

The numerical calculation program is shown in Figure 1.

The ultimate value of the bending moment was calculated relative to the compressed zone of concrete following SP 63.13330.2018 p. 8.1.8.

(1) $$M\leq M_{ult},$$

where *M* is the ultimate bending moment.

The value of $M_{ult}$ for bent elements of the rectangular section at $\xi =\frac{x}{h_{0}}\leq \xi_{R}$ is determined by the formula:

(2) $$M_{ult}=R_{b}\cdot b\cdot x\cdot \left(h_{0}-0.5x\right)+R_{sc}\cdot A_{S}^{\prime }\cdot \left(h_{0}-a^{\prime }\right)$$

where $R_{b}$ is the calculated value of the resistance of concrete to compression, MPa; *b* is beam width, m; *x* is the height of the compressed zone, m; $h_{0}$ is the design section of the beam, m; $R_{SC}$ is the design resistance of reinforcement to compression, MPa; $A_{S}^{\prime }$ is the area of longitudinal compression reinforcement in the cross-section, m^2^; *a*′ is the distance from the resultant forces in the longitudinal compression reinforcement to the nearest edge of the section (0.02 m).

Here, the height of the compressed zone *x* is determined by the formula:

(3) $$x=\frac{R_{S}\cdot A_{S}-R_{SC}\cdot A_{S}^{\prime }}{R_{b}\cdot b}$$

where $A_{S}$ is the area of longitudinal tensile reinforcement in cross-section, m^2^; $R_{S}$ is the design value of the tensile strength of the reinforcement, MPa.

The boundary height of the compressed zone is determined by the formula:

(4) $$\xi_{R}=\frac{x_{R}}{h_{0}}=\frac{0.8}{1+\frac{\varepsilon_{s,el}}{\varepsilon_{b2}}}$$

where $\varepsilon_{s,el}$ is the relative deformation of the tensile reinforcement under stresses; $\varepsilon_{b2}$ is relative deformation of concrete.

(5) $$\varepsilon_{s,el}=\frac{R_{S}}{E_{S}}$$

The values of relative deformations $\varepsilon_{b2}$ for heavy, fine-grained and tension concrete are taken with a short-term load for concrete of compressive strength class B60 and below of $\varepsilon_{b2}$ = 0.0035.

The calculation of rectangular sections was carried out depending on the height of the compressed zone according to Formula (3):

(a) For $\xi =\frac{x}{h_{0}}\leq \xi_{R}$—from the condition $M<R_{b}\cdot b\cdot x\cdot \left(h_{0}-0.5x\right)+R_{sc}\cdot A_{S}^{\prime }\cdot \left(h_{0}-a^{\prime }\right)$, where *ξ* is the relative height of the compressed zone of concrete;
(b) For $\xi >\xi_{R}$—from the condition $M<\alpha_{R}\cdot R_{b}\cdot b\cdot h_{0}^{2}+R_{SC}\cdot A_{S}^{\prime }\cdot (h_{0}-a^{\prime })$, where $\alpha_{R}=\xi_{R}\left(1-0.5\xi_{R}\right)$.

#### 2.2.2. Experimental Test Method

**Figure 2:**
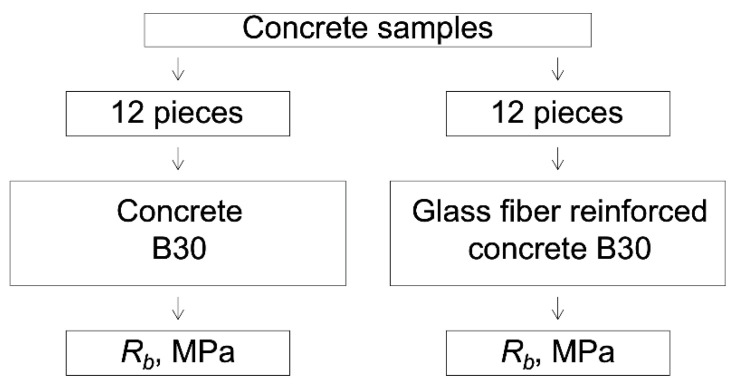
Laboratory research program.

The laboratory research program is shown in Figure 2.

The concrete mixture was made in a laboratory concrete mixer BL-10 (OOO “ZZBO”, Zlatoust, Russia). First, the dry components were mixed for 60 s; then, the mixture was mixed with water and mixed until a homogeneous consistency was obtained. The fiber-reinforced concrete mixture was prepared according to the method described in [52].

Compressive strength tests of specimens “were carried out in accordance with GOST 10180 Concretes. Methods for strength determination using reference specimens” [60] on an “IP-1000 hydraulic press (OOO NPK TEHMASH, Neftekamsk, Russia)”. “All samples of one series were tested at the age of 28 days for no more than 1 h. Loading of the samples was carried out continuously with a constant rate of load increase until its destruction. In this case, the loading time of the sample until its destruction was at least 30 s. The maximum force achieved during the test was taken as the breaking load. Cube specimens were installed with one of the selected faces on the lower support plate of the press centrally relative to its longitudinal axis, using the marks made on the press plate. The sample was loaded to fracture at a constant rate of load increase (0.6 ± 0.2) MPa/s”. The compressive strength of concrete was determined with an accuracy of 0.1 MPa using the formula:

(6) $$R=\alpha \frac{F}{A}$$

where *F* is the breaking load, N; *A* is the area of the working section of the sample, mm^2^; α is a scale factor for converting the strength of concrete to the strength of concrete in samples of basic size and shape (for cubes with an edge size of 100 mm, it is 0.95).

The strength of concrete in a series of samples was determined as the arithmetic mean of the strength of the tested samples in a series of six samples, of which four samples had the highest strength.

In total, 4 series of concrete samples were made and tested. Two series are control samples without fiber, and the rest are samples reinforced with glass fiber (in one series, there are 6 cube samples). That is, in total, 24 sample cubes of two types of concrete were destroyed (12 of each type).

The microstructure of samples with fibers was studied using a ZEISS CrossBeam 340 “microscope equipped with an Oxford Instruments X-Max 80 X-ray microanalyzer (Carl Zeiss Microscopy GmbH (Factory), Jena, Germany)” [52,61].

In addition, the study used testing and auxiliary equipment as well as measuring instruments used earlier in [51,52,61,62,63,64,65,66,67,68].

## 3. Results

### 3.1. Numerical Calculation Results

**Figure 3:**
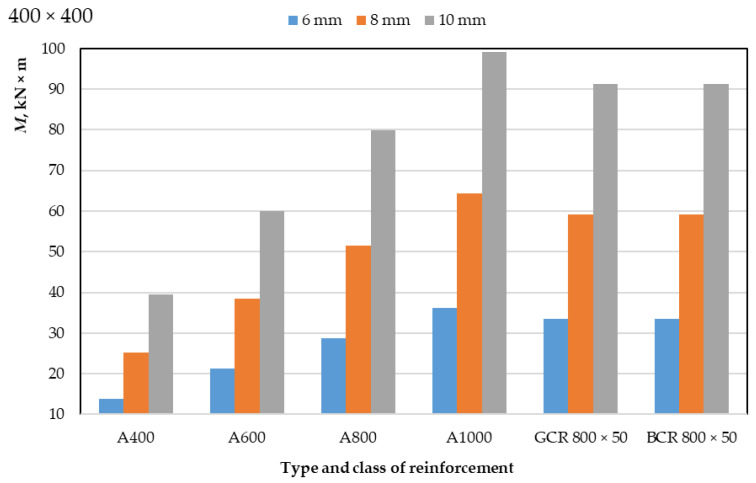
Dependence of the ultimate bending moment of concrete reinforced beams with a cross-section of 400 mm × 400 mm on the type, class, and diameter of the reinforcement.

**Figure 4:**
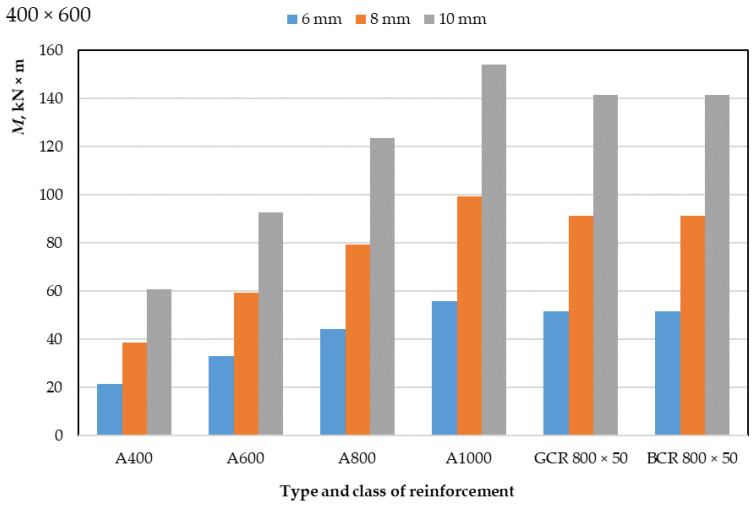
Dependence of the ultimate bending moment of concrete reinforced beams with a cross-section of 400 mm × 600 mm on the type, class, and diameter of the reinforcement.

**Figure 5:**
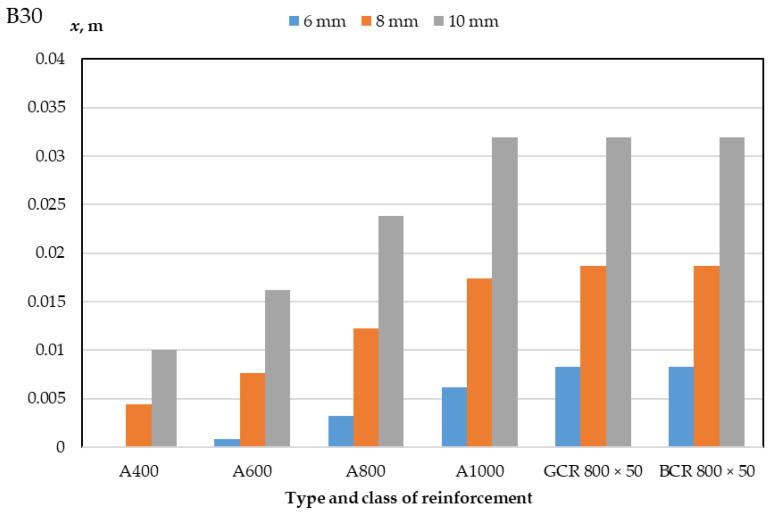
Dependence of the height of the compressed zone of concrete of class B30-reinforced beams on the type of class and the diameter of the reinforcement.

**Figure 6:**
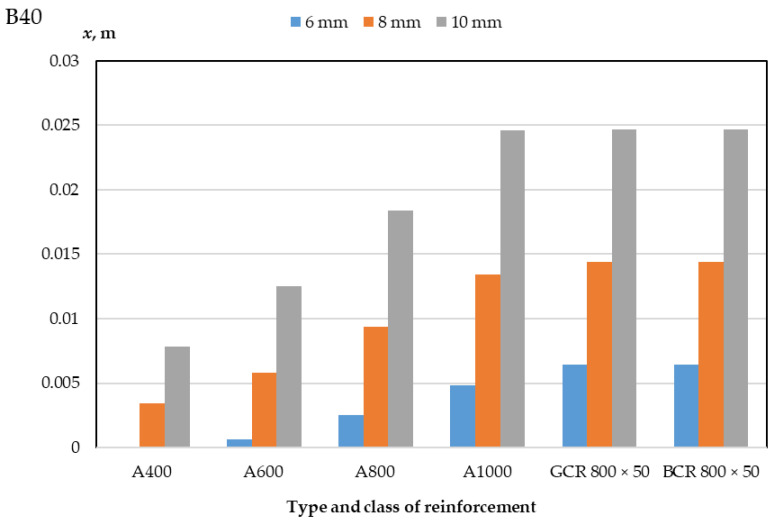
Dependence of the height of the compressed zone of concrete of class B40-reinforced beams on the type of class and the diameter of the reinforcement.

The results of a numerical calculation of the height of the compressed zone of concrete of classes B30 and B40 and the limiting value of the bending moment of reinforced concrete beams with a cross-section of 400 mm × 400 mm and 400 mm × 600 mm with a diameter of reinforcement bars of 6 mm, 8 mm, 10 mm are presented in Table 3 and Table 4 and in Figure 3, Figure 4, Figure 5 and Figure 6. In the experiment, the size of the beam section, the class of concrete, and the type, class, and diameter of the reinforcement were varied.

As a result of a numerical calculation of the limiting value of the bending moment of reinforced concrete beams, as presented in Table 3 and Table 4 and in Figure 3 and Figure 4, it can be seen that:

(1) The values of the bending moment of beams reinforced with polymer composite rebar are between the values of the moment of beams with steel reinforcement of class A800 and class A1000, slightly closer to A1000 reinforcement; this applies to each rebar diameter applied (6, 8 and 10 mm);
(2) The values of the bending moment of beams reinforced with glass-composite and basalt-composite reinforcement do not differ;
(3) The values of the limiting bending moment do not change for different classes of concrete but depend on the dimensions of the section of the elements;
(4) The ultimate bending moment of 400 mm × 600 mm beams is approximately 50% greater than that of 400 mm × 400 mm beams for each applied reinforcement diameter;
(5) When replacing 6 mm reinforcement with 8 mm reinforcement, the ultimate bending moment of the beam increases by 77–82%, while an increase in the diameter of the rods from 8 to 10 mm leads to an increase in the bending moment by 54–58%;
(6) An increase in the class of steel reinforcement from A400 to A600 leads to an increase in the ultimate bending moment of beams by 51–55%, from A600 to A800 by 32–35%, from A800 to A1000 by 24–26%;
(7) The ultimate bending moment of members reinforced with GCR 800 × 50 and BCR 800 × 50 polymer composite rebar is 130–160% greater than that of members reinforced with A400 class steel rebar, 50–58% greater than with rebar A600, 14–17% more than with A800 rebar and 7–9% less than with A1000 rebar; this difference depends on the diameter of the reinforcement and the dimensions of the section of the element and is about 50%.

Thus, the efficiency of bending elements with polymer composite reinforcement is practically comparable to the efficiency of bending elements with A1000 class steel reinforcement.

As a result of a numerical calculation of the height of the compressed zone of concrete of bending elements, as presented in Table 3 and Table 4 and in Figure 5 and Figure 6, it can be noted that:

(1) The height of the concrete compression zone of beams reinforced with polymer composite reinforcement exceeds the height of the concrete compression zone of beams with class A1000 steel reinforcement for bar diameters of 6 and 8 mm and is practically comparable to the values of the concrete compression zone of beams with class A1000 steel reinforcement for a diameter of 10 mm;
(2) The values of the height of the compressed zone of concrete beams reinforced with glass–composite and basalt–composite reinforcement do not differ;
(3) The values of the height of the compressed zone of concrete do not change with different sizes of the section of the elements but depend on the class of concrete used;
(4) The compression zone height of B40 concrete is 28–32% less than the compression zone height of B30 concrete for each applied reinforcement diameter;
(5) When replacing reinforcement with a diameter of 6 mm for reinforcement of 8 mm, the height of the compressed zone of the beam concrete increases by 125% for polymer composite reinforcement and up to 850% for A600 steel reinforcement, while an increase in the diameter of the rods from 8 to 10 mm leads to an increase in height compressed zone by 70% for PCR and 130% for A400;
(6) An increase in the class of steel reinforcement depending on the diameter of the rods from A400 to A600 leads to an increase in the height of the compressed zone from 62% to 73%, from A600 to A800 leads to an increase from 47% to 300%, and from A800 to A1000 leads to an increase from 34% to 94%;
(7) The height of the concrete compression zone of elements reinforced with GCR 800 × 50 and BCR 800 × 50 polymer composite rebar is 220–325% greater than that of elements reinforced with A400 class steel rebar, 97–967% greater than with fittings A600, 34–160% more than with fittings A800, and 0–34% more than with fittings A1000; this difference depends on the diameter of the reinforcement and the class of concrete and is about 28–32%.

### 3.2. Experiment Test Results

**Figure 7:**
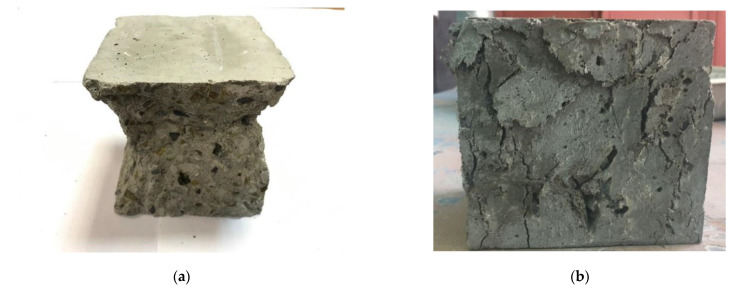
The nature of the destruction of samples of (**a**) concrete without fibers; (**b**) fiber concrete.

**Figure 8:**
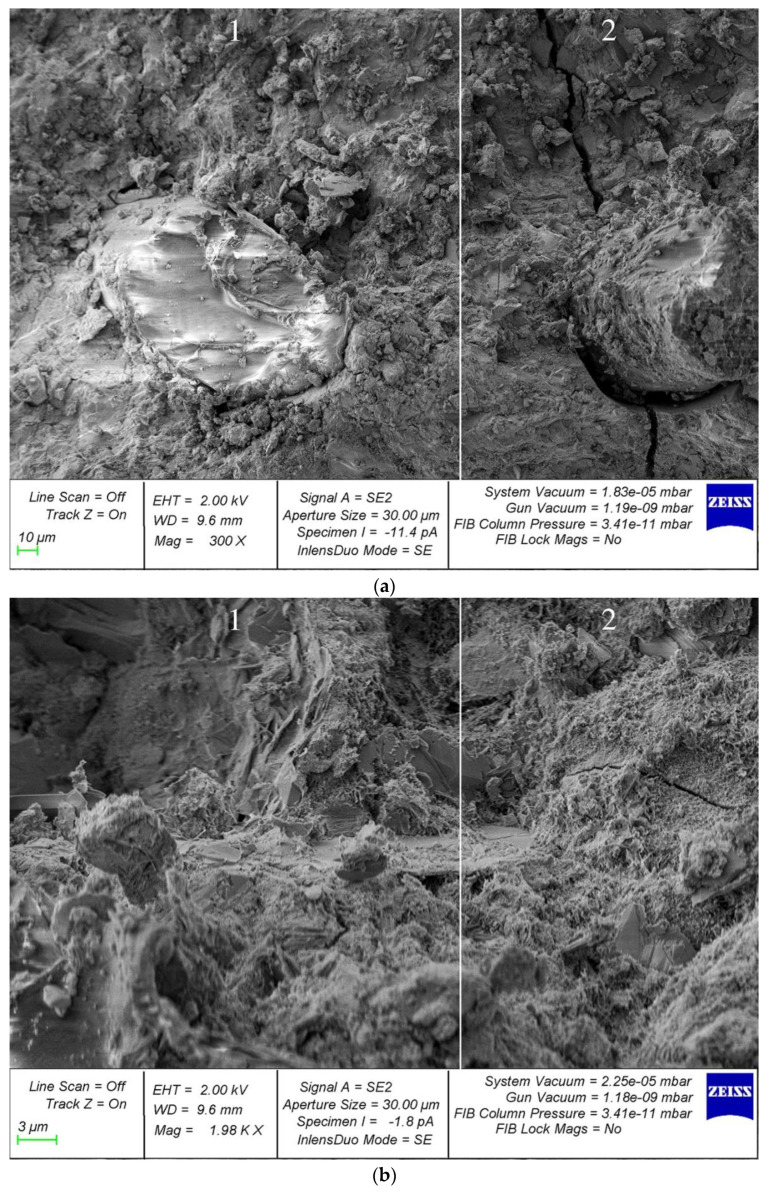
Microstructure of samples reinforced with glass fiber with magnification: (**a**) 300×; (**b**) 2000×.

The results of the laboratory experiment are presented in Table 5 and in Figure 7 and Figure 8.

Table 5 shows that due to the rationally selected glass fiber concrete mixture [51,52,61], it is possible to increase the class of concrete from B30 to B40, while the compressive strength of concrete increases up to 20%. The nature of the destruction of the sample in the form of a symmetrical pyramid demonstrates the optimality of the applied formulation and technology with a uniform distribution of properties in the body of the sample (Figure 7a). Fiber gives the concrete a more ductile fracture behavior (Figure 7b) and also improves the uniformity characteristics of the concrete. For example, as can be seen from Table 5, the standard deviation of the compressive strength values for glass fiber-reinforced concrete is almost two times less than for the same concrete but without fiber.

According to the results of experimental data, a synergistic effect was revealed, which manifests itself in an increase in the characteristics of the resulting bent reinforced elements due to the combined reinforcement with dispersed fiber and polymer composite reinforcing bars. Thus, we studied the parameters, some of which did not reveal specific dependencies and, thus, did not give grounds to consider them as an opportunity to obtain a synergistic effect. These are such characteristics as the relative height of the compressed zone and the boundary relative height of the compressed zone. Let us dwell in more detail on the height of the compressed zone *x* and the limiting value of the bending moment *M*. Thus, according to the results of numerical calculation of improved combined-reinforced elements, a synergistic effect was revealed, which was found during the calculations and was subject to interpretation in the form of universal dependencies, which are given below.

So, knowing that for standard-reinforced elements without improving the characteristics of concrete, whether it be polymer composite reinforcement or other reinforcement, for example, steel, we have a simple dependence of the form:

(7) x=f(B)+f(A)

where *x* is the height of the compressed zone, *B* is the class of concrete, and *A* is the class of reinforcement.

The indicated dependence is canonical and is applied under the existing norms and rules for the calculation and design of building structures. However, in the bending elements proposed by us with an improved microstructure and improved characteristics, we are dealing with a new type of elements obtained based on the same technological and design templates.

However, as revealed as a result of a numerical calculation and physical experiments, the height of the compressed zone, like other characteristics, varies, and, depending on various factors, from an insignificant degree to a significant one. Thus, we believe that now we are dealing with an indicator of not the height of the compressed zone *x* but the height of the reduced zone, that is, $x{,}_{red}$. Thus, in this dependence, the coefficient *k* derived by us arises—a controlled significant coefficient of the synergistic efficiency of the combined reinforcement, and the dependence takes the form:

(8) $$x{,}_{red}=f_{1}\left(B\right)+f_{2}\left(A\right)+k\left(B\cdot A\right)$$

where *k* is a controlled significant coefficient of synergistic efficiency of combined reinforcement.

Similarly, the dependence for determining the moments of bending reinforced concrete elements changes; that is, the indicator by which its bearing capacity and operational reliability are evaluated. In addition, in standard calculations and technological processes, designers put *M* and its dependence in the form:

(9) $$M=f_{3}\left(B\right)+f_{4}\left(A\right)$$

We introduce again our assumptions, which were confirmed by numerical and physical experiments; then, *M* is transformed into $M{,}_{red}$, and the formula becomes:

(10) $$M{,}_{red}=f_{3}\left(B\right)+f_{4}\left(A\right)+k\left(B\cdot A\right)$$

where *k* is a controlled, significant coefficient of synergistic efficiency of combined reinforcement.

In this case, it is necessary to distinguish between the coefficients *k*, assigning to them the indices of those indicators, the function of which we find. Thus, *k* for the height of the compressed zone will look like $k_{x}$, and the coefficient *k* for the ultimate bending moment will look like $k_{M}$.

Let us present a practical example based on experimental data, in particular, for one of the cases shown in the tables above.

$$k_{x}=\frac{X{,}_{red}-X}{X}\cdot 100\%=\frac{0.0064-0.0008}{0.0008}\cdot 100\%=700\;k_{M}=\frac{M{,}_{red}-M}{M}\cdot 100\%=\frac{33.504-21.272}{21.272}\cdot 100\%=57$$

Thus, we have proved numerically and experimentally the synergistic effect arising from the combined reinforcement of glass fiber polymer composite concrete bending elements and also presented universal dependencies for their determination. The ranges of the coefficients *k* lie in the region of the gains obtained for the height of the compressed zone and moments and range from 7 to 967. Of course, the controllability of this coefficient is ensured by the set of factors and recipe–technological parameters that are set during the design.

For a deeper analysis of the effectiveness of the effect of fiber on the structure of the composite, SEM analysis of samples with fiber was carried out. The microstructure of the sample reinforced with glass fiber is shown in Figure 8.

Analysis of photographs of the microstructure of fiber-reinforced composite samples revealed different types of destruction. In particular, in Figure 8a area 1, there are no microcracks in the region of the fiber; the structure looks homogeneous and defect-free. This is confirmed by the larger increase in area 1 in Figure 8b. However, in some areas, where the fiber reinforcement is thicker, cracks appear, propagating from the points of contact between the fiber and the cement composite (Figure 8a,b, region 2). Therefore, an important factor is the rational uniform distribution of the fiber dosage carefully selected for each type of concrete, which reduces cracking at the points of contact of the fiber with the cement composite and, accordingly, improves the structure and characteristics of fiber-reinforced concrete.

Thus, in addition to the formulation and design aspects of obtaining fiber-reinforced concrete composites with improved characteristics, the technological aspect is also important, which consists in carefully selected modes of preparation of the fiber-reinforced concrete mixture and, in particular, the rational distribution of fiber in the volume of the concrete matrix [51,52,61].

### 3.3. Economic Analysis Results

**Figure 9:**
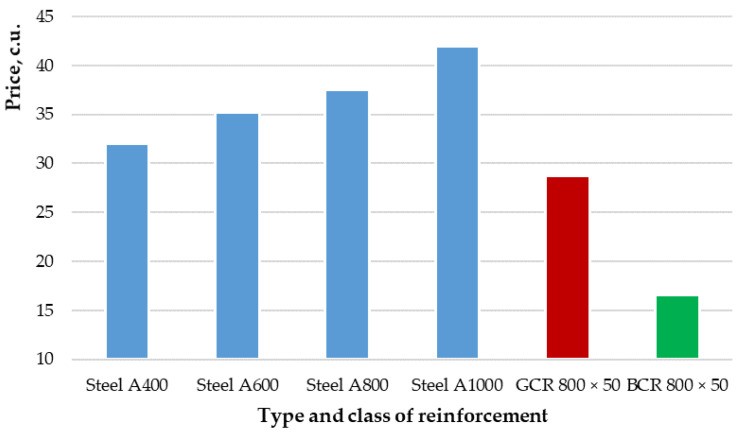
Diagram of the average cost of various types and classes of reinforcement using the example of a diameter of 8 mm.

In addition, an analysis of the cost of the types and classes of reinforcement considered in the study was carried out using the example of a diameter of 8 mm. Data taken from various catalogs of rebar sellers in Russia made it possible to compile the average cost per unit of production. The results of the analysis are presented in Table 6 and Figure 9.

Table 6 and Figure 9 show that the cost of basalt-composite reinforcement (green column in Figure 9) is two to three times less than the cost of steel reinforcement of classes A400, A600, A800 and A1000 (blue columns in Figure 9). At the same time, glass–composite reinforcement (red column in Figure 9) is 70% more expensive than basalt–composite, but it is cheaper than steel from 11 to 50%, depending on the reinforcement class. This confirms the economic efficiency in terms of the market value of polymer composite reinforcement compared to steel.

## 4. Discussion

It is known that the most traditional type of reinforcement for reinforced concrete elements operating in bending is reinforcing steel of various grades. However, with all the demand for traditional reinforced concrete, it has several disadvantages, the main of which should be highlighted: the high cost of steel rods and the heavy weight of the resulting structures. In addition, at present, there are effective types of polymer composite reinforcement, such as glass composite reinforcement and basalt composite reinforcement, which make it possible to obtain bendable reinforced elements from heavy concrete with even more efficient work in structures.

In this regard, we made a market analysis, which revealed that the cost parameters of basalt–composite reinforcement are the most preferable. At the same time, the cost of basalt–composite reinforcement, ceteris paribus, is almost two times less than glass-composite reinforcement, and their bearing capacities practically do not differ from each other. Thus, in our opinion, the best way to obtain the most efficient and rational building structures from heavy concrete, working in bending, should be made from GFPCC.

Fiber reinforcement, as we have proved earlier [51,52,61,62,63], allows us to increase the class of concrete, other things being equal, on a significant scale. Despite the fact that the class of concrete is not the main characteristic that increases the operational reliability of bending elements made of heavy concrete, it still plays its role in achieving the quality and reliability of such a design. Thus, considering the results obtained by us earlier, we expect to obtain such complex structures while achieving a synergistic effect in terms of improving the efficiency of work, reducing the cost, and reducing the weight of the resulting structures.

Let us consider and analyze the results we have achieved from three points of view. First, it is necessary to identify the effect that we achieved in the study in the prescription aspect. In particular, we have proved that fiber reinforcement increases the characteristics of concrete by 10–20% percent and improves its microstructure in accordance with [51,52], while the microstructure receives a denser packing of particles and, due to a more rational distribution of fiber fibers, microcrack formation near concentration of fibers in the body of the concrete matrix. In this regard, in comparison with the works of the authors [9,17,18,19,20,21,22,23,24,25,26,27], we obtained higher results in terms of strength and also achieved an improved microstructure of the material. All this is proposed for use not only in compressed elements, as in [18,19,28,29,30,31,32,33], but, of course, in tensioned bending elements, which emphasizes the more rational operation of fiber-reinforced elements, since fiber reinforcement is primarily directed for bending and tensile loads. In this regard, our result surpasses the results of [6,9,14,17,18,27,33,34,35,36,37,38].

**Figure 10:**
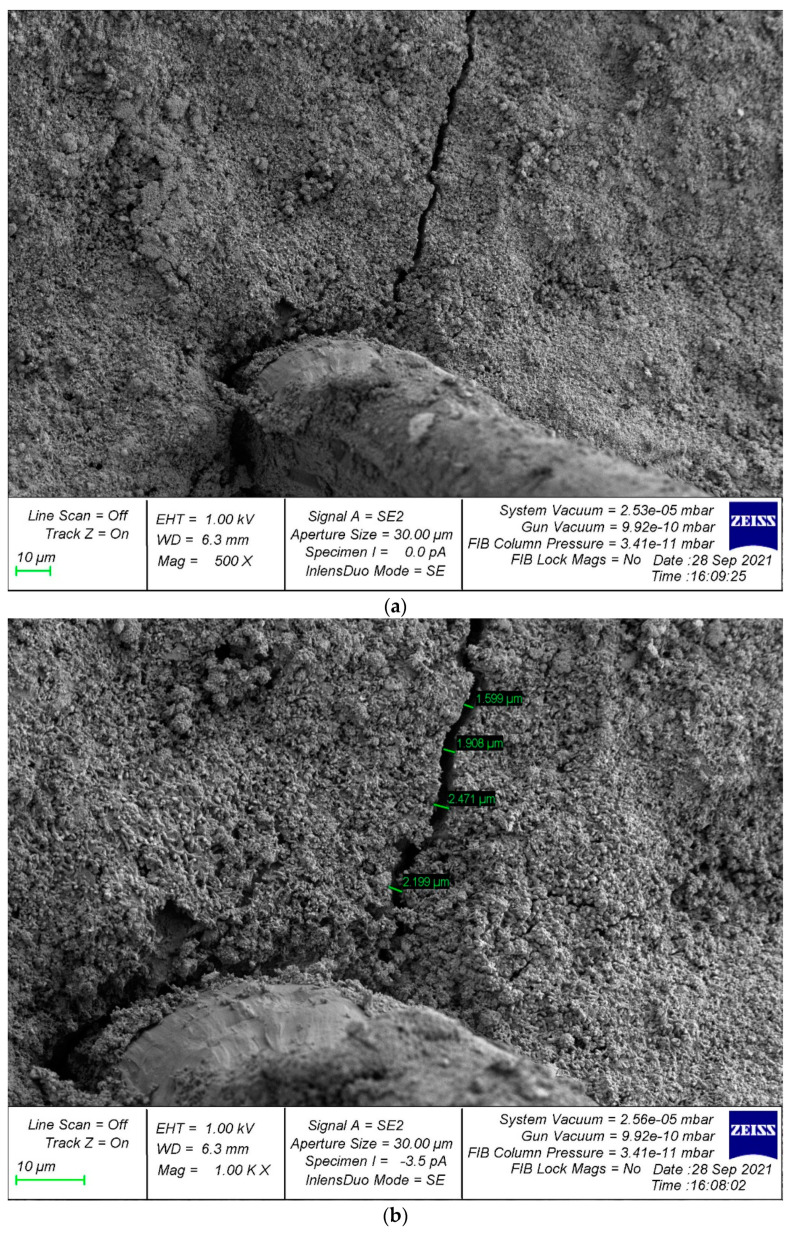
Analysis of crack formation in the “fiber–contact zone–cement matrix” system with magnification: (**a**) 500×; (**b**) 1000×.

As for microstructural studies of fiber reinforcement, the obtained results of SEM analysis are in good agreement with the results obtained by us earlier in [61]. On the surface of the cement matrix of polydisperse-reinforced samples, a smaller number of microcracks formed during the destruction of prototypes is observed. In addition, polydisperse-reinforced ones are characterized by a smaller opening width of microcracks. The average width of their opening in polydisperse-reinforced samples is significantly lower than in monodisperse-reinforced samples (Figure 10) [61].

**Figure 11:**
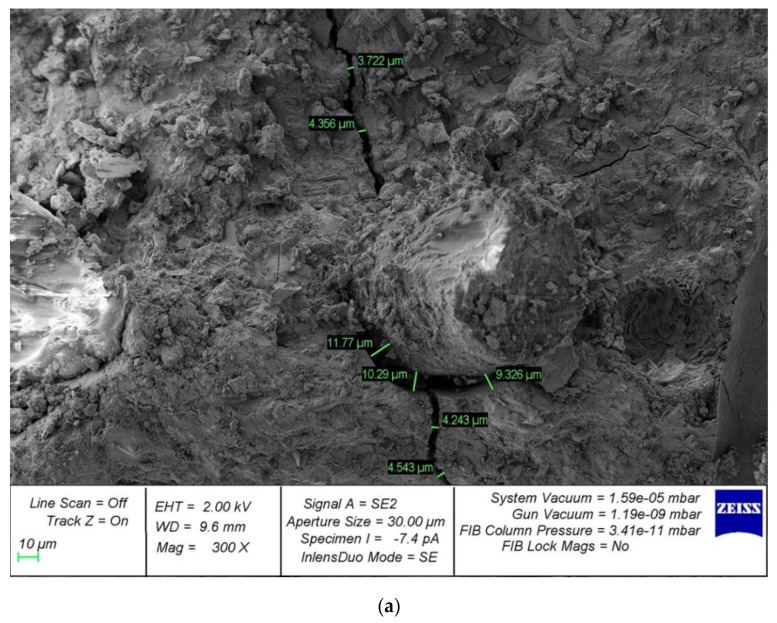

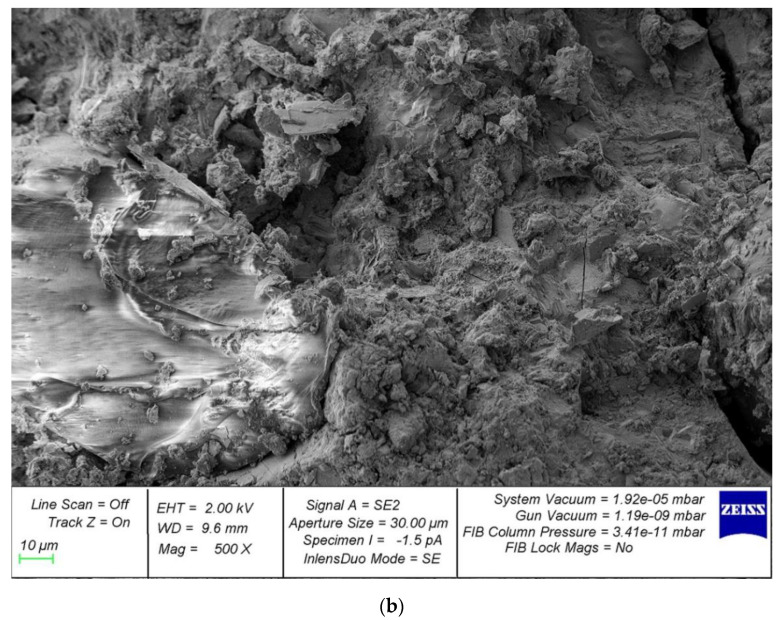
Photographs of the microstructure of the fiber-reinforced sample with magnification: (**a**) 300×; (**b**) 500×.

The analysis of photographs of the microstructure carried out also continues and develops the results already obtained by us earlier in [51]. The nature of the development of cracks directly depends on the rational distribution of fiber fibers in the body of the cement matrix. In areas that allow us to evaluate the usefulness of the rational distribution of fibers, there are no microcracks in the region of the fibers. However, with improper homogenization and distribution of the fiber-reinforcing fiber, around the formed fiber bundles, defects appear at the same time, expressed in microcracks, which in principle correlates with the analogue of this phenomenon at the macro level—excessive density of reinforcement of complex structures. Thus, microscopic studies confirm the thesis not only about the initial characteristics and quality of the fibers used but also about the importance of their distribution and homogenization in the body of the matrix, since microcracking occurs already at this stage and can develop at the macro level (Figure 11) [51].

Next, consider the constructive effect we have achieved, it consists of improving the performance of an improved design due to the synergistic effect of fiber reinforcement of bending elements and in combination of such dispersed reinforcement with polymer composite reinforcement with rods. In this regard, our results seem to be effective in comparison with the results of the authors [1,2,3,4,5,6,7,8,9,10,11,12,13,14,15]. Our results showed a higher bearing capacity and especially in combination with the fact that the mass of our elements is less than similar reinforced concrete elements.

Finally, from a price point of view, the cost of our elements is reduced and is more economical, as confirmed by our economic analysis. This is in good agreement with the results of the authors [1,2,3,4,5,6,7,8,9,10,11,12,13,14,15,16,35,37,38,39,40,41,42,43,44,45,46,47,48,49,50].

Thus, focusing on the comparison of experimental data and calculated data with the results of other authors, we note the advantages of our proposals, which are justified by the proven synergistic effect. In addition, the analysis of the results revealed the relationship between the initial parameters and components and technological, constructive and economic indicators of the structures being created.

## 5. Conclusions

(1) Theoretically and experimentally substantiated the effectiveness of combined-reinforced glass fiber polymer composite concrete bending elements from the point of view of three aspects: prescription, technological and constructive.
(2) The improvement of the structure and characteristics of glass fiber-reinforced concrete and glass fiber polymer composite concrete bending elements of a new type has been proven; the compressive strength of glass fiber-reinforced concrete with careful observance of rational parameters in terms of formulation and technology has been increased to 20%, the efficiency of glass fiber polymer composite concrete bending elements is comparable to the work of reinforced concrete bending elements with steel reinforcement class A1000 and higher.
(3) Analytical dependences of the type of functions of the characteristics of bent concrete structures on the arguments are proposed—the parameters of combined reinforcement with fiber and polymer composite reinforcement; the synergistic effect of such development is described, and a new coefficient *k* is proposed—a controlled significant coefficient of the synergistic efficiency of the combined reinforcement.
(4) From a price point of view, the cost of the developed elements is reduced and is economically more profitable (up to 300%), which is confirmed by the economic analysis.

The prospects and direction of development of the research are planned in terms of bending elements made of heavy concrete with a differentiation of characteristics over the section as well as with the use of differentiated fiber reinforcement with fibers from various materials.

## Figures and Tables

**Table 1:** Physical and mechanical characteristics of glass fiber.

Density, g/cm^3^	Tensile Strength, GPa	Elastic Modulus, GPa	Fiber Length, mm	Elongation, %
2.6	1.8	70	12	1.5

**Table 2:** Properties of the applied reinforcement.

Characteristics		Steel A400	Steel A600	Steel A800	Steel A1000	GCR	BCR
$\mathrm{Estimated}\;\mathrm{value}\;\mathrm{of}\;\mathrm{tensile}\;\mathrm{strength}\;R_{s}$, MPa		340	520	695	870	800	800
$\mathrm{Design}\;\mathrm{compressive}\;\mathrm{strength}\;R_{sc}$, MPa		340	470	500	500	300	300
$\mathrm{Modulus}\;\mathrm{of}\;\mathrm{elasticity}\;E_{s}$, MPa		200				50	50
$\mathrm{Area}\;\mathrm{of}\;\mathrm{longitudinal}\;\mathrm{tensile}\;\mathrm{reinforcement}\;\mathrm{in}\;\mathrm{section}\;A_{s}$, mm^2^	Ø6	113.0					
	Ø8	201.0					
	Ø10	314.0					
$\mathrm{Relative}\;\mathrm{deformation}\;\mathrm{of}\;\mathrm{tensile}\;\mathrm{reinforcement}\;\mathrm{at}\;\mathrm{stresses}\;\mathrm{equal}\;\mathrm{to}\;R_{s}$ $,\;\varepsilon_{S,el}\times 10^{-3}$		1.7	2.6	3.48	4.35	16	16

Note: Steel A400—steel reinforcement class A400; Steel A600—steel reinforcement class A600; Steel A800—steel reinforcement class A800; Steel A1000—steel reinforcement class A1000 [56]; GCR—glass composite reinforcement; BCR—basalt composite reinforcement [57].

**Table 3:** Results of a numerical calculation of the design characteristics of bending elements with a section size of 400 × 400 mm.

Product Section Size, mm	Concrete Class	Product Length, mm	Reinforcement			Boundary Relative Height of the Compressed Zone, $\varepsilon_{R}$	Height of Compressed Zone *x*, m	Relative Height of the Compressed Zone ε	Ultimate Bending Moment M, kN × m
			Type	Class	Diameter, mm				
400 × 400	B30	3000	steel	A400	6	0.5385	0	0	13.836
					8		0.0044	0.0116	25.13
					10		0.01	0.0264	39.457
			steel	A600	6	0.459	0.0008	0.0022	21.272
					8		0.0076	0.0199	38.453
					10		0.0162	0.0426	60.092
			steel	A800	6	0.4014	0.0032	0.0085	28.688
					8		0.0122	0.0322	51.435
					10		0.0238	0.0626	79.874
			steel	A1000	6	0.3567	0.0062	0.0162	36.112
					8		0.0174	0.0458	64.278
					10		0.0319	0.0838	99.226
			GCR	800 × 50	6	0.1436	0.0083	0.0219	33.451
					8		0.0187	0.0491	59.23
					10		0.0319	0.0841	91.306
			BCR	800 × 50	6	0.1436	0.0083	0.0219	33.451
					8		0.0187	0.0491	59.23
					10		0.0319	0.0841	91.306
	B40		steel	A400	6	0.5385	0	0	13.836
					8		0.0034	0.0089	25.145
					10		0.0078	0.0204	39.535
			steel	A600	6	0.459	0.0006	0.0017	21.272
					8		0.0058	0.0154	38.497
					10		0.0125	0.0329	60.294
			steel	A800	6	0.4014	0.0025	0.0066	28.696
					8		0.0094	0.0249	51.55
					10		0.0184	0.0484	80.311
			steel	A1000	6	0.3567	0.0048	0.0125	36.141
					8		0.0134	0.0354	64.512
					10		0.0246	0.0648	100.011
			GCR	800 × 50	6	0.1436	0.0064	0.0169	33.504
					8		0.0144	0.0379	59.499
					10		0.0247	0.065	92.095
			BCR	800 × 50	6	0.1436	0.0064	0.0169	33.504
					8		0.0144	0.0379	59.499
					10		0.0247	0.065	92.095

**Table 4:** Results of a numerical calculation of the design characteristics of bending elements with a section size of 400 × 600 mm.

Product Section Size, mm	Concrete Class	Product Length, mm	Reinforcement			Boundary Relative Height of the Compressed Zone, $\varepsilon_{R}$	Height of Compressed Zone *x*, m	Relative Height of the Compressed Zone ε	Ultimate Bending Moment M, kN × m
			Type	Class	Diameter, mm				
400 × 400	B30	3000	steel	A400	6	0.5385	0	0	21.523
					8		0.0044	0.0076	38.795
					10		0.01	0.0173	60.809
			steel	A600	6	0.459	0.0008	0.0014	33.028
					8		0.0076	0.0130	59.353
					10		0.0162	0.0279	92.748
			steel	A800	6	0.4014	0.0032	0.0056	44.4
					8		0.0122	0.0211	79.368
					10		0.0238	0.041	123.52
			steel	A1000	6	0.3567	0.0062	0.0106	55.781
					8		0.0174	0.03	99.245
					10		0.0319	0.0549	153.862
			GCR	800 × 50	6	0.1436	0.0083	0.0143	51.537
					8		0.0187	0.0322	91.384
					10		0.0319	0.0551	141.546
			BCR	800 × 50	6	0.1436	0.0083	0.0143	51.537
					8		0.0187	0.0322	91.384
					10		0.0319	0.0551	141.546
	B40		steel	A400	6	0.5385	0	0	21.523
					8		0.0034	0.0059	38.81
					10		0.0078	0.0134	60.887
			steel	A600	6	0.459	0.0006	0.0011	33.028
					8		0.0058	0.0101	59.397
					10		0.0125	0.0216	92.95
			steel	A800	6	0.4014	0.0025	0.0043	44.408
					8		0.0094	0.0163	79.484
					10		0.0184	0.0317	123.957
			steel	A1000	6	0.3567	0.0048	0.0082	55.810
					8		0.0134	0.0232	99.479
					10		0.0246	0.0424	154.647
			GCR	800 × 50	6	0.1436	0.0064	0.0111	51.591
					8		0.0144	0.0249	91.653
					10		0.0247	0.0426	142.335
			BCR	800 × 50	6	0.1436	0.0064	0.0111	51.591
					8		0.0144	0.0249	91.653
					10		0.0247	0.0426	142.335

**Table 5:** Values of compressive strength of the studied types of concrete.

Type of Concrete	Compressive Strength, MPa
Concrete B30	41.9 ± 2.3
Glass fiber-reinforced concrete B30	50.3 ± 1.8

**Table 6:** The average cost of a unit of reinforcement with a diameter of 8 mm.

Num.	Type and Class of Reinforcement	Amount	Cost in Conventional Units
1	Steel A400	1 m	32.0
2	Steel A600	1 m	35.2
3	Steel A800	1 m	37.5
4	Steel A1000	1 m	42.0
5	GCR 800 × 50	1 m	28.7
6	BCR 800 × 50	1 m	16.6

## Data Availability

The study did not report any data.
